# Does the Maternal Serum IgG Level during Pregnancy in Primary Antibody Deficiency Influence the IgG Level in the Newborn?

**DOI:** 10.1155/2015/286380

**Published:** 2015-08-27

**Authors:** Vasantha Nagendran, Noel Emmanuel, Amolak S. Bansal

**Affiliations:** ^1^Epsom and St Helier University Hospitals NHS Trust, Carshalton, Surrey SM5 1AA, UK; ^2^St Georges University of London, London SW17 0RE, UK; ^3^St George's University Hospitals NHS Foundation Trust, Tooting, London SW17 0QT, UK

## Abstract

*Purpose*. To find out if the serum IgG level in the newborn baby was affected by low maternal serum IgG during pregnancy in two newly diagnosed primary antibody deficient patients. *Method*. Infant cord blood IgG level was compared with maternal IgG level in 2 mothers with newly diagnosed primary antibody deficiency, who declined replacement IgG treatment during pregnancy. *Results*. Both mothers delivered healthy babies with normal IgG levels at birth. *Conclusions*. The normal IgG levels and sound health in these 2 babies in spite of low maternal IgG throughout pregnancy raise interesting discussion points about maternofoetal immunoglobulin transport mechanisms in primary antibody deficiency.

## 1. Introduction

It is conventional wisdom that the foetus and neonate depend on transplacental transfer of IgG antibodies from the mother for immune protection, until maturation of their own humoral immunity. IgG is the only immunoglobulin class that is significantly transferred across the human placental barrier. The crossing of IgG from the mother to foetus occurs throughout pregnancy via endosomes within the syncytiotrophoblasts of the placenta, through a pH dependent mechanism involving FcRn receptors, with a possible role for other IgG Fc receptors, yet to be fully elucidated. The transfer is influenced by the maternal IgG and specific antibody concentrations, IgG subclass, gestational age, placental integrity, and the nature of the antigen, being more intense for thymic dependent antigens [1].

IgG transport to the foetus begins mainly in the second trimester of pregnancy and is maximal in the third trimester. IgG1 subclass is transported most efficiently and IgG2 least efficiently, and transfer of pneumococcal antibodies appears to be serotype specific and IgG subclass dependent [2, 3].

Common variable immunodeficiency (CVID) and hyper IgM syndrome (HIGM) which was previously included under the umbrella of CVID are heterogeneous primary antibody deficiency states characterised by low IgG and impaired antibody responses. Management of both of these conditions involves replenishing the IgG deficiency and monitoring and treating infections, autoimmune diseases, and other complications. Immunoglobulin replacement therapy (RIT) may be administered by intravenous (IVIg) or subcutaneous (SCIg) routes.

If pregnant mothers with CVID or HIGM had no RIT, placental transfer of IgG may be reduced, causing a deficiency of protective antibodies to defend the foetus from intrauterine infections. This lack of passive immunity may also increase the risk of infection in the child's first few months of life, until its immune system matures.

There are presently no published protocols on the management of CVID or HIGM patients during pregnancy. CVID mothers receiving intravenous immunoglobulin (IVIg) therapy are believed to transfer exogenous IgG through the placenta in similar patterns as endogenous immunoglobulins, and therefore mothers are advised to continue regular RIT (by intravenous or subcutaneous route) and encouraged to breast-feed their babies [4–8].

The management of CVID or HIGM that is* first* diagnosed during pregnancy is more complicated and has* not *hitherto been addressed in the literature. We report our experience of 2 women in this situation who declined replacement immunoglobulin therapy during their pregnancy.

## 2. Case Reports

### 2.1. Patient 1

A 40-year-old woman pregnant with her second child was seen for recurrent upper respiratory tract infections. She had suffered a series of colds and bouts of productive cough after the birth of her first child, born by Caesarian delivery at term, 3 years earlier. At 20 years of age, she had required 6 months of oral prednisolone therapy for low platelets.

Blood tests at 9 weeks of gestation confirmed a total IgG of 2.5 g/L (normal 6.0–16.0), IgM of 1.22 g/L (0.5–1.9), and IgA of <0.056 g/L (normal 0.8–2.8). There was no paraprotein on immunoelectrophoresis. Her B cell count was normal at 0.17 × 109/mL and she had normal numbers of CD4 and CD8 T cells.

She was advised to start immunoglobulin therapy for CVID, but, in spite of detailed discussions with medical staff on several occasions, she was reluctant to do this until after she had delivered the child by elective Caesarian delivery. The patient remained well during pregnancy and had only minor upper respiratory tract infections, not requiring antibiotic therapy.

A healthy female baby weighing 3150 gm was born at term and cord blood analysis detailed in Table 1 showed a total IgG 5.5 g/L (cord blood normal range: 5.2–18.0 g/L), IgA <0.18 g/L, and IgM <0.23 g/L. IgG subclasses showed IgG1 4.07 g/L, IgG2 0.13 g/L, IgG3 0.51 g/L, and IgG4 <0.083 g/L. Pneumococcal Abs at 2 *μ*g/mL and Hib Abs at 0.03 *μ*g/mL were sub-therapeutic according to accepted values [9–11], but the level was satisfactory for tetanus toxoid at 0.43 IU/mL [12, 13]. Numbers of CD19 B cells and CD4 T cells were normal but CD8 T cells were slightly reduced. The child was breast-fed and remained well in infancy. Repeat serum immunoglobulin assessment at one year of age showed normal levels.

The patient's Caesarian scar became infected and required antibiotics. She commenced regular IVIg therapy 2 months later following detailed investigations for specific antibody production after vaccination. CT scan of the chest showed mild bronchiectasis but pulmonary function tests were essentially normal. Approximately 6 years from delivery, she was relatively free of infections but had a relapse of idiopathic thrombocytopenia (ITP) and also developed autoimmune haemolytic anemia (AIHA).

### 2.2. Patient 2

A 38-year-old lady was investigated for suspected parvovirus infection in the first trimester of her second pregnancy as 4 children in the nursery where she worked were thought to have “slapped cheek” virus infection. Her results showed previous exposure and immunity (IgG antibody to parvovirus, but no IgM antibody). However, her serum immunoglobulins IgG was found to be low. She had been previously well with no significant sinus, ear, chest, or gastrointestinal infections. Her first pregnancy was uneventful, and she had delivered a healthy baby boy.

Our initial investigations in the first trimester confirmed low total IgG 4.3 g/L (normal: 6.0–16.0), low IgA 0.56 (normal: 0.8–2.8), and high IgM 8.32 g/L (normal: 0.5–1.9). The immunoelectrophoresis did not detect any paraprotein to account for this profile. Serum IgG subclass measurements showed low IgG 1 (3.07 g/L) and normal IgG2, IgG3, and IgG4. The pneumococcal antibodies and* Haemophilus* antibodies were low, and tetanus antibodies were normal. There was lymphopenia with low T and B cell counts and low CD4 and CD8 subset counts. The B cell proportion was high (35%) with slight decrease in T cell proportion (54%) and normal CD4-CD8 ratio. CVID classification is as follows: Warnatz Class = 1a, Piqueras Class = MB1, and EuroClass = SmB (−) Tr(norm)21(low). The autoantibody screen, RF, ANCA, TPO, and ACA (IgG) were negative, but ACA IgM was slightly elevated at 13.1.MPLU/mL (normal: 0–9.8).

She was well in herself and very reluctant to embark on any further investigations (for the high IgM) or new treatments, so we arranged to monitor her for infections and check serum immunoglobulins during her pregnancy. Although she remained well, repeat serum IgG in the third trimester was lower (3.6 g/L), IgM remained elevated at 8.16 g/L, and IgA was 0.57 g/L. The lymphopenia noted in the first trimester had reverted to normal. We discussed that maternofoetal transfer of immunoglobulins occurred in the third trimester of pregnancy and strongly advised immunoglobulin replacement. After a detailed discussion of the pros and cons with medical staff, she made an informed decision to consider this only* after* the birth of her baby. She had a full term normal delivery of a healthy female baby weighing 3570 grams and cord blood analysis showed a total IgG 9.2 g/L (cord blood normal range: 5.2–18.0 g/L), IgA <0.18 g/L, and IgM <0.23 g/L. There was insufficient sample to measure IgG subclasses and specific antibodies.

Mother breast-fed her baby, from birth. She postponed her postpartum clinic visit but confirmed that she and her baby were in good health. Blood tests arranged through her general practitioner 10 months after the birth showed low IgG (5.2 g/L), low IgA (0.7), and elevated IgM (10.5 g/L) in the mother, who confirmed her good health but declined further investigations for HIGM. The baby was infection-free and thriving, and her serum immunoglobulins at 10 months were normal (IgG 5.07 g/L, IgM 0.38 g/L, and IgA 0.18 g/L). Mother has moved away from our area and has not attended for follow-ups.

## 3. Discussion

We present two women discovered to have low IgG during their second pregnancy. The first had experienced chest infections over the preceding three years and had previous treatment for presumed idiopathic thrombocytopenia. The second patient had no history of infections. Both mothers had informed discussions with the consultant immunologists about RIT. The importance of maternofoetal immunoglobulin transfer to protect the foetus and the newborn baby and the potential of blood products (including IVIg) to transmit hitherto unidentified infections (e.g., prions) were discussed. Both mothers opted to defer RIT until after they delivered their babies. In spite of low maternal IgG levels, both delivered healthy babies with normal cord blood total IgG. Patient 1 commenced RIT 2 months after delivery. Patient 2 chose to defer RIT, as she was symptom-free and her serum IgG was 5.2 g/L 10 months postpartum (compared to 3.6 g/L in the third trimester).

The cord/maternal IgG ratios noted in our antibody deficient women were within the 0.75 to 2.86 range reported in healthy women [14]. It is interesting that the ratios were similar in both mother/baby pairs despite significantly different total maternal IgG levels, and the transfer ratio was slightly higher in patient 1 who had lower levels of maternal IgG, confirming an active transfer process to maintain foetal IgG levels.

Specific IgG transfer ratios could only be calculated for patient 1 due to insufficient sample in newborn 2. Here, the transfer ratios of specific IgG antibodies to tetanus and pneumococcal capsular polysaccharides were preserved, but the transfer ratio was significantly reduced for* Haemophilus influenzae* b (Hib).

Importantly both of our babies remained well and infection-free after birth, with normal serum immunoglobulins, and neither appears to have suffered infection with either Hib or* S. pneumoniae.*

Our findings raise interesting questions about physiological mechanisms that may operate to maintain normal foetal IgG levels when maternal levels are low. Observations that neonatal cord blood antibody level is higher than maternal levels date back to several decades but were disregarded or attributed to measurement error. In 1966, a study from Scripps Clinic, California, using more “advanced methods” concluded that cord blood IgG was significantly higher than in the mother and attributed this to active placental transport [15]. In 1968, a Finnish study found significantly higher titres of IgG antibodies in full-term cord sera compared to maternal titres, in 7 out of 12 antibodies studied [16]. Other studies showed that low maternal values did* not* necessarily predict equally low levels in cord blood; in fact, they tended to exceed the maternal level [17]. An inverse relationship between maternal and foetal levels of IgG antibodies to herpes simplex, tetanus toxoid, streptolysin O, and* S. pneumoniae* was reported in 1996 [18].

We speculate that an “upregulation” of FcRn and other IgG Fc receptors in the endosome of the syncytiotrophoblast may help increase the transport of IgG from mother to foetus, when maternal IgG is low. This may explain why both of our patients delivered babies with normal IgG and high IgG transfer ratios, in spite of low IgG levels in their mothers.

Although high transfer ratios were achieved without IgG replacement therapy in our two patients, IgG level in newborn of patient 1 is considered low and could have been raised if the mother had received IVIg therapy during pregnancy, and this may have also improved her Hib level, affording optimal protection for the newborn.

The recent worldwide interest in maternal immunisation is aimed at protecting the newborn in the first few months of life (before primary immunisation becomes effective), and the basis of this is the demonstration of a strong positive correlation between maternal and infant specific antibody responses to vaccine-preventable infections such as pertussis, tetanus,* Haemophilus*, and* pneumococcus* [19]. Mothers with primary antibody deficiency states (e.g., CVID and HIGM) are unable to respond effectively to immunisations and must therefore rely on immunoglobulin infusions (IVIg or SCIg) to boost their levels of specific antibodies. In a detailed study of two mothers with IVIg treated CVID and their newborns, it was demonstrated that cord blood IgG levels were greater than maternal IgG; subclasses IgG1, IgG3, and IgG4 were preferentially transferred compared with IgG2; anti-protein (tetanus) IgG antibodies were equivalent to or higher than maternal levels with good transfer of polysaccharide (Hib) IgG antibodies; and anti-*Streptococcus pneumoniae* avidity indices were similar between mothers and their neonates [6].

Therefore, in primary antibody deficiency states, the potential benefit of immunoglobulin therapy to both the pregnant mother and the baby cannot be overemphasised.

## 4. Conclusion

Women with hypogammaglobulinemia can deliver healthy infants with normal IgG levels, but careful monitoring of both mother and baby is important. Further research is required to fully understand transplacental transfer of protective IgG antibodies in the presence of low maternal serum IgG levels.

## Figures and Tables

**Table 1:** Summary of serum/cord blood total IgG and specific IgG levels against pneumococcal polysaccharides, *H. influenzae* b, and tetanus toxoid in patients and their babies.

Test (units)	Patient 1	Baby of patient 1	Transfer ratio	Patient 2	Baby of patient 2	Transfer ratio
IgG (g/L)	2.5^*∗*^	5.5^*∗∗*^	2.20	4.3^*∗*^	9.2^*∗∗*^	2.14
Pneumococcal antibody ug/mL (protective level >20 ug/mL)	2.0	2.0	1.0	8.0	Insufficient sample	—
*H. influenzae* antibody ug/mL (protective level >0.15 ug/mL)	0.90	0.03	0.033	0.10	Insufficient sample	—
Tetanus antibody u/mL (protective level >0.01 u/mL)	0.31	0.43	1.39	0.24	Insufficient sample	—

^*∗*^Normal range of serum IgG for adults: 6.0–16.0 g/L.

^*∗∗*^Normal range of cord blood IgG: 5.2–18.0 g/L.
